# The promise of anti-ErbB3 monoclonals as new cancer therapeutics

**DOI:** 10.18632/oncotarget.550

**Published:** 2012-08-10

**Authors:** Luigi Aurisicchio, Emanuele Marra, Giuseppe Roscilli, Rita Mancini, Gennaro Ciliberto

**Affiliations:** ^1^ Takis s.r.l., Via di Castel Romano 100, Roma, Italy; ^2^ BIOGEM scarl, via Camporeale, Ariano Irpino (Av), Italy; ^3^ Dipartimento di Medicina Clinica e Molecolare, Facoltà di Medicina e Psicologia, Università degli Studi di Roma “La Sapienza”, Roma; ^4^ IRCCS Istituto Nazionale Tumori, Fondazione “G. Pascale”, Napoli, Italy; ^5^ Dipartimento di Medicina Sperimentale e Clinica, Università degli Studi di Catanzaro“Magna Graecia”, Catanzaro, Italy

**Keywords:** cancer, ErbB3, monoclonal antibodies, mechanism of action, clinical trials

## Abstract

In the last 3-5 years strong evidence has been gathered demonstrating ErbB3 as a key node for the progression of several cancer types. From the mechanistic standpoint the intracellular region of this receptor is rich of tyrosine residues that, upon phosphorylation, become high affinity binding sites for PI3K and other proteins involved in signal transduction. The involvement of ErbB3 occurs at different levels, most likely as a consequence of its promiscuity in the interaction with other RTKs of the same or other families. Several efforts are therefore being put in the development of antibodies that target this receptor either singly or in combination with other synergizing receptors. Some of these compounds have already entered clinical development. Although clinical proof-of-concept has not yet been achieved, this is likely to occur soon and will further accelerate the inclusion of anti-ErbB3 monoclonals in the repertoire of anticancer agents for more effective combination therapy. In this paper we review the wealth of anti-ErbB3 antibodies under development and compare their properties and potential to become marketed drugs.

## INTRODUCTION

The EGFR family of receptor tyrosine kinases consists of four closely related family members: EGFR (Her1), ErbB2 (HER2), ErbB3 (Her3), and ErbB4 (Her4) [1]. These receptors are important regulators of normal growth and cell differentiation. Their gene amplification, overexpression or mutation is associated with tumor development and poor clinical prognosis in most of the human cancers [2]. These cell surface receptors are characterized by a composite extracellular domain which contains a well defined ligand-binding site in at least three of the four members (EGFR, ErbB3 and ErbB4), a single pass transmembrane domain, followed by an intracellular domain where a tyrosine kinase domain and a C-terminal non-catalytic signaling tail can be distinguished [3].

Signaling through these receptors is mediated by homodimerization or heterodimization with other family members, usually mediated by a rather promiscuous set of ligands. Multiple ligands are known to bind to the EGFR family. Some of them bind specifically to EGFR (EGF, TGF-α, and amphiregulin) or ErbB4 (neuregulin 3, neuregulin 4, and tomoregulin), whereas others have dual specificity (e.g., β-cellulin, epiregulin, and heparin-binding EGF-like growth factor for both EGFR and ErbB4, and neuregulins 1 and 2 for both ErbB3 and ErbB4) [4].

**Figure 1:**
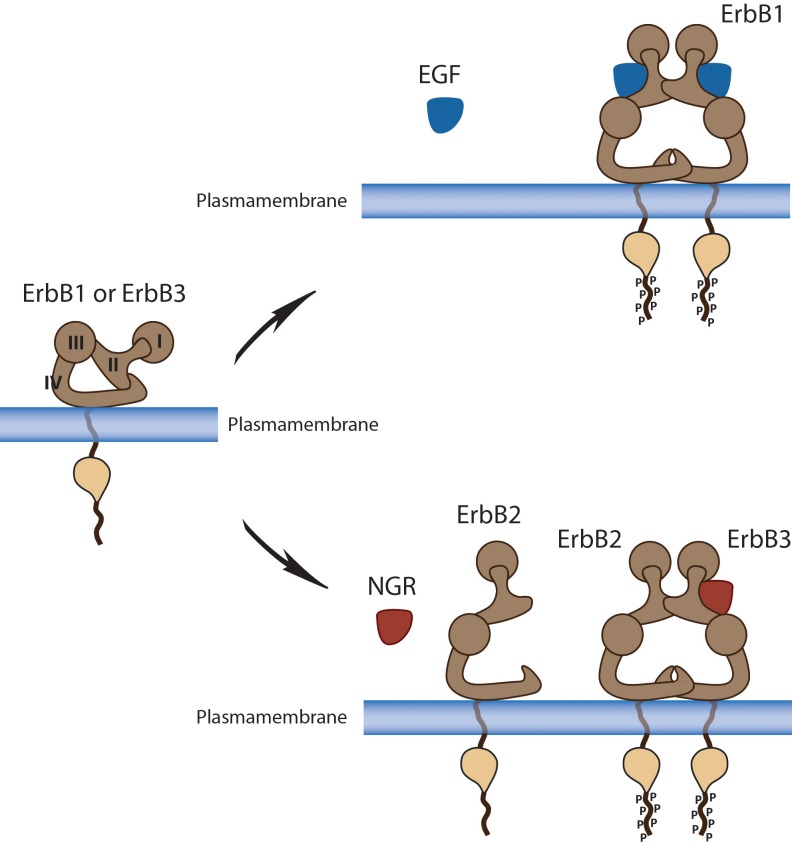
ErbB receptors ligand dependent change of conformation and signal transduction In the absence of ligand, a direct intramolecular interaction between domains II and IV keeps ErbBs in a closed (locked or tethered) conformation that prevents interaction between domains I and III. This conformation disrupts the ligand-binding pocket and buries the dimerization arm of domain II. *Upper panel:* EGFR or ErbB1. *Lower Panel:* HER2 is inherently unable to dimerize because of a strong interaction between domains I and III which leads to a constitutively extended dimerization arm. HER2 is therefore constantly primed for interactions with ligand-bound receptors of the ErbB family. In the presence of NRG, the dimerization loop from domain II of ErbB3 extends to interact intramolecularly with a ligandless, primed HER2 monomer to form the oncogenic HER2-ErbB3 heterodimer.

With the exception of HER2, the extracellular domains of the EGFR family members are unable to form stable homo- or heterodimers in the absence of ligand. Binding of the ligand with the extracellular domain of its corresponding receptor induces a structural reconfiguration, which promotes the exposure of the otherwise tethered dimerization domain; this allows the formation of dimers which results in receptor transphosphorylation of tyrosine residues within the activation loop leading to enhanced kinase activity with subsequent phosphorylation of tyrosine residues in the carboxy-terminal domain. This in turn enables recruitment of signaling molecules and activation of downstream intracellular signaling pathways [5] (Fig 1). The EGFR family members can also be activated through ligand-independent activation. These mechanisms include activation by non-physiological stimuli (e.g., oxidative stress, UV, and γ-irradiation) or by other receptor tyrosine kinases (notably MET, IGF-1R, or TRK-B). The importance of dimerization in EGFR family signaling is best illustrated by HER2 and ErbB3. HER2 is the preferred dimerization partner for all the EGFR family members. However, although HER2 has the strongest kinase activity, there is no known ligand to this receptor. Therefore, activation of HER2 is dependent upon dimerization with other family members [6]. In contrast, ErbB3 carries a well defined binding site for ligands but lacks intrinsic kinase activity and is therefore dependent upon heterodimerization to be phosphorylated in its signaling tail and to induce downstream signaling [6].

Activated EGFR family members recruit various adaptors and signaling molecules through the phosphorylated cytoplasmic domain, which further leads to activation of a variety of downstream signaling pathways [7]. All of the EGFR family members activate the extracellular signal-regulated kinase (Erk)1/2 via recruitment of Grb2 or Shc adaptors [8, 9]. Activation of Erk1/2 has an important role in EGFR stimulated cell proliferation. Activation of another important signaling pathway for cell proliferation and survival, the phosphatidylinositol 3-kinase (PI3K)/Akt pathway, however, differs between the EGFR family members. Whereas ErbB3 and ErbB4 are capable of binding the p85 regulatory subunit of PI3K directly through their putative p85 binding sites (tyrosine-X-X-methionine), EGFR and HER2 bind indirectly to p85 through adaptors or through heterodimerization with ErbB3 or ErbB4 [10,11]. In addition to Erk1/2 and PI3K activation, phosphorylated EGFR family members can also activate a collection of transcription factors such as c-fos, c-jun, c-myc, signal transducer and activator of transcription, NF-kB, zinc-finger transcription factor, and Ets family members.

Two EGFR family receptor members, namely EGFR itself and HER2, have been amongst the most extensively studied targets for the therapy of cancer over the past twenty years and a wealth of drugs directed against them have been either already approved or are in advanced clinical development. EGFR is being targeted with the monoclonal antibodies cetuximab and panitumumab [12-17] and with two low molecular weight tyrosine kinase inhibitors, gefitinib and erlotinib [18-23] in several types of epithelial cancers, including head and neck, pancreatic, colorectal and a subset of non small cell lung cancers with mutant or highly expressed EGFR.

HER2 is targeted by the monoclonal antibody trastuzumab (Herceptin™) in breast cancers [24, 25], where it is overexpressed in approximately 20-25% of cases and this approach is now clinically approved for the treatment of breast cancer patients expressing HER2 at high levels (+3 herceptest™). However, trastuzumab had little or no effectiveness against cancers of the prostate [26], pancreas [27], colon and rectum [28] or lung epithelia [29], which also very often express HER2 at lower levels. Other antibodies targeting HER2 are Pertuzumab, a humanized antibody recognizing a different HER2 epitope involved in homo- or heterodimerization [30] which has very recently been approved by FDA in combination with trastuzumab and docetaxel for the treatment of patients with HER2-positive metastatic breast cancer (mBC) who have not received prior anti-HER2 therapy or chemotherapy for metastatic disease; and T-DM1, a drug conjugated version of trastuzumab, which has recently demonstrated superb clinical effects in a controlled Phase II trial conducted in Trastuzumab-resistant patients [31]. Finally, lapatinib, a small molecule inhibitor of both EGFR and HER2 has been developed [32], which is currently used in the treatment of HER2^+^ metastatic breast cancer resistant to trastuzumab. [33].

ErbB3 has been disregarded for several years as a cancer target, although the elevated expression of this receptor in several human cancers led in early times to postulate its involvement in tumor progression [34]. This low interest in ErbB3 was also due to the lack of detectable mutations in cancer samples and the absence of a strongly active tyrosine kinase in its intracellular domain [35]. However, during the past 5 years a mounting number of evidences have been accumulated pointing to a key role of this receptor in tumorigenesis and cancer progression and, above all in the establishment of resistance to therapies [36]. These evidences have triggered major efforts towards the development of anti-ErbB3 therapies. Because this receptor is devoid of strong intrinsic kinase activity, the major strategy in this case is the generation of monoclonal antibodies directed against the receptor. Some of these have already entered clinical development. It is reasonable to expect therefore, that in the next years if clinical proof-of-concept is achieved ErbB3 may become a next generation blockbuster target for cancer therapies.

### Validation of ErbB3 as new cancer target

#### ErbB3 in Lung cancer

ErbB3 was shown to be highly expressed in lung adenocarcinomas and associated with poor prognosis as measured by immunohistochemistry [37]. Moreover quantitative real time indicated that high ErbB3 expression was associated with decreased survival in patients with early stage (I-IIIA) NSCLC [38]. ErbB3 was constitutively activated at high level in several lung adenocarcinoma cell lines, which also express HER2 [39]. Co-expression of ErbB3 with other ErbB family members was indicative of tumor recurrence [40]. The expression of the proliferation-associated marker Ki-67 at a higher frequency in ErbB3-positive NSCLC cases than in ErbB3-negative tumors was suggestive of a contribution of ErbB3 to an aggressive cancer behaviour [41]. Additional strong evidence for the importance of ErbB3 expression in lung tumorigenesis came from ErbB3 transgenic mice. These mice treated with the carcinogen methylnitrosourea developed a high incidence of lung tumors with reduced latency compared to wild type mice [42]. In addition mice double transgenic for human ErbB3 and rat HER2/*neu* had an incidence of spontaneous lung tumors similar to that of single transgenic mice, but with a shorter latency [42], thus demonstrating the relevance of HER2/ErbB3 heterodimers in lung tumorigenesis.

ErbB3 has also been shown to play a role in drug resistance: suboptimal pathway inhibition by tyrosine kinase inhibitors (TKIs) was shown to result in a compensatory shift in ErbB3 activation [43]. In this case ablation of ErbB3 expression by siRNAs restored efficacy and pro-apoptotic activities of TKIs [43]. Likewise, amplification of cMet was described to occur in cells resistant to TKI treatment and cMet transphosphorylation of ErbB3 was shown to be a mechanism whereby resistant cells can circumvent blockade of EGFR activity [44]. These data suggest that targeting ErbB3 could be a novel strategy to treat drug resistant tumors.

#### ErbB3 in Breast cancer

In breast cancer, increased ErbB3 expression is common. Indeed ErbB3 mRNA levels relative to normal gland and measured by real-time PCR are increased in 46% of breast cancers [45] and correlate positively with those for ErbB4 and negatively with EGFR mRNA [46, 47].

ErbB3 protein is detectable in 50–70% of human breast cancers by immunohistochemistry [48, 49]. Moreover, tyrosine-phosphorylated ErbB3 is frequently over-expressed in breast tumors that overexpress HER2 [48, 50]. This led to the suggestion that ErbB3 could be an important partner of HER2 in the development of breast tumors. It has been shown, in fact, in NIH 3T3 *in vitro* assays and in a transgenic mouse model that the heterodimer HER2-ErbB3 is able to transform cells and to induce mammary tumors [51]. Finally, Holbro and colleagues have demonstrated that HER2 inactivation blocked proliferation in HER2-overexpressing cells and this was associated with a decrease of ErbB3 phosphorylation [52].

On the other hand, there seems to be lack of clarity about the relationships between ErbB3 expression and estrogen receptor (ER). At the protein level, ErbB3 and ER did not correlate [50, 53] and a high percentage of ER-negative tumors were strongly positive for ErbB3 [50].

The ErbB3 receptor is emerging as a critical element not only in HER2-mediated transformation and tumor progression but also in drug resistance. In fact, inhibition of HER2 phosphorylation by TKIs targeting EGFR and HER2 in HER2^+^ breast cancer cells is followed by feedback upregulation of activated ErbB3 [54]. Other evidences underline the importance of ErbB3 in HER2 addicted breast cancers. In HER2-overexpressing cells, inhibitors of the PI3K pathway induce a compensatory up-regulation of the expression and phosphorylation of ErbB3 [55, 56]. Moreover, knocking down ErbB3 results in sensitization to PI3K inhibitors [57]. In breast cancer cell lines BTK474-HR20 and SKBR3-pool2 selected in vitro for resistance to trastuzumab a strong upregulation of ErbB3, pErbB3, IGF1R and pIGFR is observed which strongly contribute to cell proliferation [58]. Indeed in these cells destabilization of both ErBB3 and IGF1R by metformin exerts a strong anti-proliferative effect [58]. Finally in recent studies the group of Cook and collaborators [59, 60] has shown, using a variety of animal models, that ErbB3 ablation impairs PI3K/Akt-dependent mammary tumorigenesis and p44/42 phosphorylation in pre-neoplastic HER2-overexpressing mammary glands and tumors. This was associated with decreased growth of pre-existing HER2-overexpressing tumors and improved tumor responses to HER2 tyrosine kinase inhibitors. All together these findings suggest that ErbB3 cooperates with HER2 to induce changes in breast epithelium before, during and after tumor formation. Hence, therapeutic targeting of ErbB3 in combination with HER2 may be the most appropriate strategy to achieve full efficacy of anti-HER2 targeted therapy, in particular for breast cancer.

#### ErbB3 in Colorectal cancer

ErbB3 is occasionally mutated in colon carcinomas [61]. ErbB3 mRNA is overexpressed in the majority of human colon cancer cell lines and in human colorectal tumors [62]. Few studies have investigated the ErbB3 protein expression: the data obtained show that ErbB3 expression can be detected in 36-90% of colorectal cancer [63, 64]. ErbB3 is frequently co-expressed together with EGFR and HER2 [65]. In a recent study, colon carcinoma cells were transfected with a single chain antibody against HER2 and showed inactivation of HER2 tyrosine phosphorylation and reduced heterodimerization of HER2 with ErbB3. This reduction resulted in a increase of EGFR expression with the subsequent formation of heterodimers of EGFR with ErbB3 [66]. The signalling by EGFR-ErbB3 heterodimers may play an important role in colon cancer cell lines; in fact, inhibition of AKT phosphorylation and cell proliferation by the EGFR-specific inhibitor erlotinib (Tarceva) correlated with expression of ErbB3 [67]. As further evidence of ErbB3's importance in this type of cancer, inhibition of proliferation and induction of apoptosis in HT-29 colon cancer cells by conjugated linoleic acid may be mediated by its ability to downregulate ErbB3 signaling and PI3K/ AKT pathway [68].

#### ErbB3 in Melanoma

ErbB3 is frequently expressed in human melanoma cell lines [69] and microarray analysis revealed that ErbB3 was one of a small number of genes whose upregulation is characteristic of melanoma [70]. Immuno-histochemical analysis showed high ErbB3 expression in melanoma metastases and suggested increased ErbB3 expression associated with disease progression [71]. Moreover, activated ErbB3 has been observed in primary melanocytic lesions and this suggests that activation of NRG1/ErbB3 signaling may contribute to the progression of melanoma from benign nevi to metastatic malignancies. The role of ErbB3 in melanoma can also be indirectly inferred from the evidence [72] that driver mutations are found in ErbB4, its preferred heterodimerizing partner in these cells, in a significant percentage of melanomas (approximately 20%). These mutations can be grouped into two main categories: a) kinase activating mutations similar to those found in EGFR in NSCLC and Head & Neck cancer; b) single aminoacid mutatations in domain II or IV of the extracellular domain which affect the intramolecular contact between these two domains and induce a shift towards a constitutively open conformation of the receptor [72]. Therefore, inhibition of ErbB3 with monoclonal antibodies is expected to impair the growth promoting effect of mutant ErbB4-ErbB3 heterodimers in melanomas.

Clear cell sarcoma of soft tissue (CCSST) is a rare lesion of the musculoskeletal system affecting children and young adults with melanocytic differentiation. ErbB3 is one of the most particularly up-regulated genes in these cancers. Cell lines derived from these tumors expressed ErbB3 protein and either HER2 or ErbB4 [73]; in half of the lines ErbB3 was constitutively activated by endogenous NRG1 expression; the others were responsive to added NRG1 [74].

#### ErbB3 in Ovarian Cancer

It has been reported that ErbB3 mRNA expression, as detected by RT-PCR, is increased in a proportion of ovarian cancers [75]. Of the ovarian carcinomas, 16% of the tumors overexpressed ErbB3 protein compared to normal ovarian [76]; moreover expression of NRG was detected in the majority of ovarian carcinomas and cell lines and this could be a potential for autocrine regulation of cell growth [77]. It has recently been confirmed that there was a direct correlation between ErbB3 overexpression and poor overall prognosis [78].

In OVCAR8 xenograft mouse model, down-regulation of ErbB3 by RNAi decreased tumor progression compared to controls, in the same mouse model treatment with a monoclonal anti-ErbB3 antibody also resulted in inhibition of tumour progression [79].

Signaling activation by ErbB3 in ovarian cancers seems to depend in some cases upon the formation of heterodimers with HER2 while in others, the activation of ErbB3 is independent of heterodimers with HER2 or EGFR. Interestingly, four alternate c-ErbB3 transcripts (1.6, 1.7, 2.1 and 2.3 kb) were isolated from an ovarian carcinoma-derived cell line. Fibroblasts transfected with all four transcripts express truncated ErbB-3 products. Three of these four products are soluble secreted proteins [80].

### Targeting ErbB3: Anti-ErbB3 antibodies

A wealth of antibodies targeting human ErbB3 has been generated in the past years. In this section we will review their biochemical/biological properties and current status of development. Interestingly while the majority of these agents recognize only ErbB3, a subset of them has been engineered thanks to new technologies, to be able to bind not only ErbB3 but also an additional co-receptor. A comprehensive list of such antibodies is listed in table 1 and described in the following sections.

**Table 1:** List and properties of anti-ErbB3 antibodies under development

Antibody name	Company	Isotype	Affinity for human ErbB3	Other receptors recognized	Receptor internalization and degradation	ADCC Activity	Stage of development
Monospecific							
AMG-888	U3 Pharma/Amgen/Daiichi-Sankyo	ND - fully human	ND		ND	ND	Phase II
MM-121	Merrimack/Sanofi Aventis	IgG2 - fully human	0.75 nM	mErbB3	yes	ND	Phase II
GE-huMab-HER3	Roche	IgG1 - humanized	ND		ND	Strong	Phase I
AV-203	Aveo Pharmaceuticals	IgG1 - humanized	0.076 nM	cynoErbB3	ND	ND	Phase I
TK-A3	Takis	IgG1 - humanized	7.2 nM	mErbB3	yes	ND	PreC
TK-A4	Takis	IgG1 - humanized	2.2 nM		yes - strong	ND	PreC
MP-RM-1	Mediapharma	ND	32 nM		yes	ND	PreC
LJM716	Novartis and Sanofi Aventis	ND? - fully human	ND		ND	ND	PreC
REGN1400	Regereron	ND? - fully human	0.05 nM		ND	ND	PreC
Bispecific							
MEHD7945A	Genentech	IgG1?- fully human	0.4 nM	hEGFR	ND	yes	Phase I
MM-111	Merrimack	HSA linked to scFv	10 nM	hHER2	ND	ND	Phase I
MM-141	Merrimack	HAS linked to svFv	ND	hIGF1R	ND	ND	PreC

Legend: ND: Not disclosed;cyno, cynomologous monkey; m, mouse; h, human

### Monospecific anti-ErbB3 antibodies

#### MM-121

**Figure 2:**
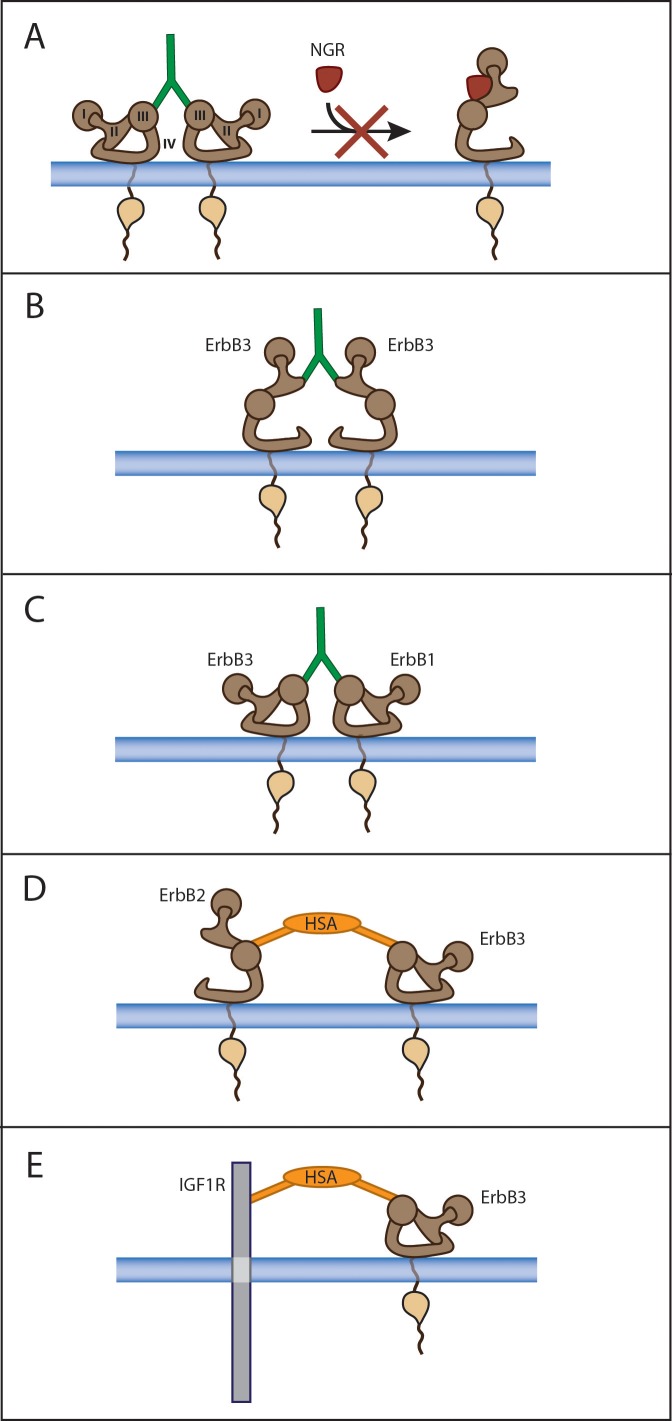
Mode of action of monospecific and dual specificity anti-ErbB3 antibodies A) a monospecific antibody binding to domain III inhibits ligand binding and blocks the receptor in a tethered configuration unable to form heterodimers with other receptors. B) the antibody binds to the heterodimerization loop located in domain II. C) a dual specificity antibody binds to domain III of two distinct receptors, such as ErbB3 and EGFR. D) a dual specificity antibody interacts with its two scFv domains simultaneously with both HER2 and ErbB3. E) a dual specificity antibody against ErbB3 and IGF1R. HSA: human serum albumin.

MM-121, a fully human IgG2 mAb that binds specifically to ErbB3, was isolated by selection from a phage display Fab library and screening for high-affinity binders by surface plasmon resonance [81]. MM-121 is being developed by Merrimack in partnership with Sanofi-Aventis. This antibody has a KD of approx 0.75 nM for human ErbB3, is cross reactive with mouse ErbB3 and competes for heregulin-1β binding to the receptor. The precise binding epitope of MM-121 to ErbB3 has not been disclosed, but the antibody is able, via a direct or indirect mechanism, to block ligand–induced receptor heterodimerization with HER2 (Fig.2). MM-121 inhibits HRG-1-induced ErbB3 and AKT phosphorylation with IC_50_ values of 2.4 and 6.0 nM respectively and completely inhibits *in vivo* tumor xenografts of renal ACHN cells. Further studies have subsequently shown through the analysis of a panel of several cell lines of various origin, that MM-121 is able to affect tumor growth only in a subset of them [82]. Sensitive tumors were further subdivided into strongly and partially responsive. In the attempt to identify biomarkers of responsiveness, the authors assessed several parameters such as ligands and ErbB receptor levels, and found that, although efficacy did not correlate with a single parameter, the best single feature capable to predict responsiveness was HRG-β1 expression. Therefore, MM-121 is mainly active in cancers with ligand-dependent activation. MM-121 effects were also assessed in a mouse model of lung cancer caused by doxycycline-induced over-expression of a double mutant EGFR T790M-L858R. This model mimics resistance to TKIs that develops in patients following the secondary mutation T790M and is characterized by increased heregulin expression and phosphorylation of ErbB3. MM-121, which is cross-reactive to the mouse homologue, was poorly active when administered as monotherapy, but exterted strong tumor growth inhibitory effect when given in combination with cetuximab [82]. MM-121 is currently being assessed in several Phase I and II clinical trials in breast cancer, non-small cell lung cancer, ovarian cancer and other solid tumor cancers.

#### AMG 888

AMG 888 (also known with the name U3-1287) is a fully human anti ErbB3 monoclonal isolated from U3 Pharma using Amgen's Xenomouse® technology. It is currently being developed by Amgen in partnership with Daiichi–Sankyo. AMG 888 was reported to block ErbB-induced AKT and ERK signaling and to inhibit *in vitro* and *in vivo* growth of multiple tumor cell lines as single agent or, even better in combination with other ErbB family inhibitors, such as cetuximab. However, the exact biochemical properties of the antibody have not been disclosed in detail [83].

Also AMG 888 is currently undergoing clinical trials. Phase I data have been reported at the 2011 ASCO meeting [84]. The phase I trial had 2 parts: based on positive tolerability and Pharmacokinetic (PK) data in part 1, a part 2 dose-expansion was conducted on 31 cancer patients with advanced solid tumors the majority of whom (no.17) were NSCLC cancer patients, at doses of 9, 14 and 20 mpk. PK data showed that steady state was reached after 3 dosing cycles and C_min_ was >10-fold greater than the threshold required to achieve 90% inhibition of ErbB3 phosphorylation in xenograft studies. Eight patients experienced stable disease. Of these one patients had approximately 26% tumor shrinkage and a partial metabolic response monitored by FDG-PET. Based on these highly encouraging data AMG 888 has been brought into Phase II clinical development.

#### TK-A3 and TK-A4

**Figure 3:**
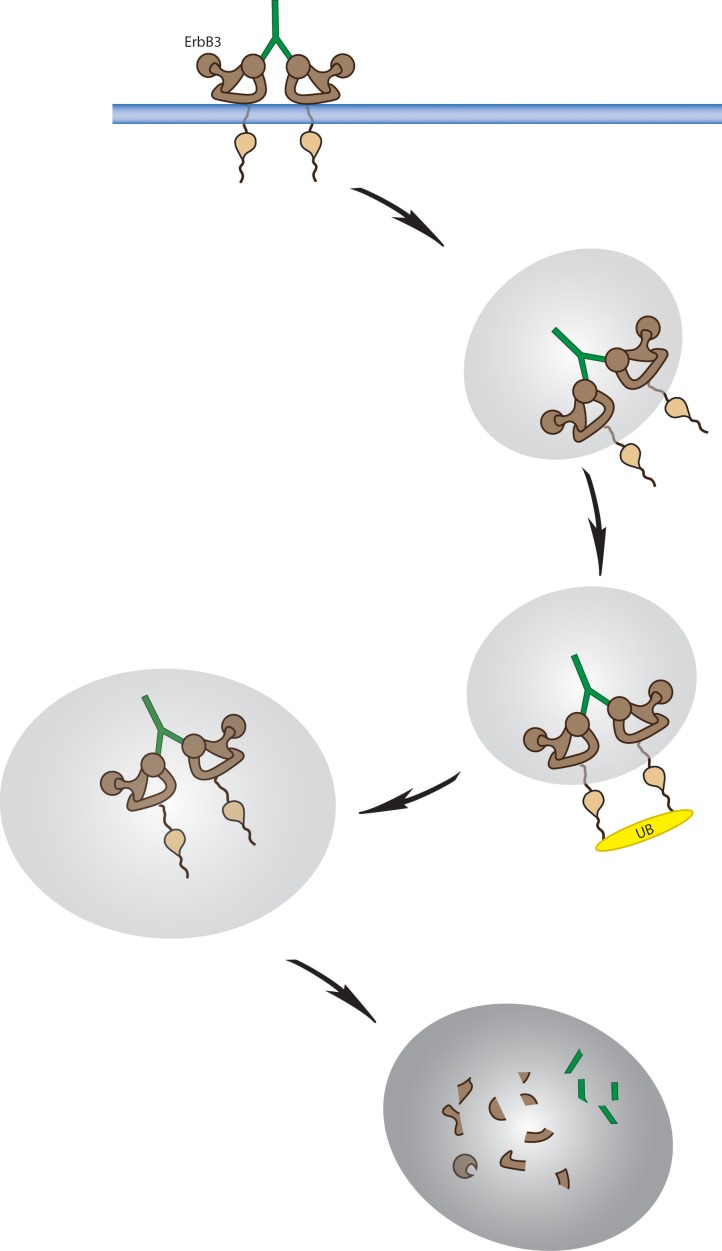
Internalization and degradation as therapeutic action by anti-ErbB3 antibodies A hypothetical model of ErbB3 endocytosis and intracellular sorting mediated by anti-ErbB3 antibodies. Antibody binding to ErbB3 leads to receptor engagement, inhibition of phosphorylation and induced sorting of the ErbB3 receptor into lysosomes. ErbB3 receptor polyubiquitination and lysosomal degradation is the final step.

TK-A3 and TK-A4 are both mouse monoclonals of IgG1a isotype generated by Takis in collaboration with the University of Catanzaro (Italy), through muscle electroporation with the human ErbB3 cDNA carrying the H584F mutation which gives rise to a constitutively extended conformation of the receptor in the absence of ligand [85]. TK-A3 and A4 show an affinity for human ErbB3 of 7.2 and 2.2 nM respectively as measured by Biacore; they block ligand binding to the receptor with an IC_50_ of 1.5 and 2.2 nM respectively and inhibit pErbB3 ligand-dependent stimulation with an IC_50_ of 4 and 6 nM respectively. *In vitro*, these two anti-ErbB3 antibodies modulate the growth rate of cancer cells of different origins and *in vivo* they show antitumor properties in several xenograft models. TK-A3 but not TK-A4 is cross reactive against the murine ErbB3 receptor. Therefore its efficacy was assessed in the HER2-driven carcinogenesis genetically engineered BALB/*neuT* mouse model for mammary tumor. In this model TK-A3 significantly slowed down tumor growth, increase time-to-disease progression and downregulated *in vivo* ErbB3-mediated signaling [85]. TK-A3 and TK-A4 mechanism of action has been studied in greater detail in two melanoma cell lines. It was shown through a series of combined cell biology approaches that antibody efficacy strongly correlated with antibody-induced receptor internalization, degradation and inhibition of receptor recycling to the cell surface (Fig3) [86]. Epitope mapping studies using a peptide array scanning approach allowed to define the epitope bound by TK-A3 but not the one recognized by TK-A4. Interestingly, TK-A3 recognized the dimerization loop in domain II of the receptor extracellular region [86]. The availability of two monoclonals directed against different epitopes of the ErbB3 extracellular domain allows to assess the potential synergy of combined treatments. Indeed it has been previously shown that combination of pairs of mAbs recognizing distinct epitopes in HER2, exert a superior anti-tumor effect than the use of individual antibodies [87]. Since the effect was observed both *in vivo* and *in vitro*, it is likely that this is not due to the involvement of immunological mechanisms but to a more powerful downregulation of the signalling survival pathways of HER2 expressing cancer cells exerted by the antibodies combinations. Humanization of TK-A3 and TK-A4 has been achieved, with maintenance of similar affinity for ErbB3 receptor and biological properties such as inhibition of NRG induced signalling and cell proliferation (our unpublished data).

#### GE-huMab-HER3

GE-huMab-HER3 is a humanized and glycoengineered IgG1 antibody that is being developed by Roche [88]. Also this antibody prevents ligand binding and receptor heterodimerization. It inhibits ligand-dependent ErbB3 and Akt phosphorylation with an IC_50_ of approximately 0.1 μg/ml). GE-huMab-HER3 efficacy *in vivo* was assessed on a panel of primary NSCLC xenografts, where it caused >50% tumor growth inhibition in 10 out of 17 NSCLC cell lines and in some cases even resulted in complete tumor remission. A unique feature of GE-huMab-HER3 that differentiates it from all other anti-ErbB3 antibodies is its ability to bind to human FcγRIIIa on immune effector cells with a 50-fold higher affinity than standard IgG1 antibodies. Therefore, GE-huMab-HER3 has superior potency and efficacy in ADCC, both in *in vitro* assays and in *in vivo* models. This property may confer to GE-huMabHER3 superior clinical efficacy, but also potentially a higher degree of toxicity. Phase I clinical testing of this antibody has recently started.

#### AV-203

AV-203 is a humanized immunoglobulin IgG1 kappa antibody that is being developed by Aveo Pharmaceuticals [89]. AV-203 shows a very high affinity for human ErbB3 (Kd = 76 pM at 37°C), is cross-reactive to cynomolgous monkey ErbB3 but not to mouse ErbB3. AV-203 is a potent inhibitor of ligand-dependent ErbB3 activation and its downstream signaling molecule Akt, blocks ErbB3/HER2 heterodimerization and moderately down regulates ErbB3 receptor *in vitro* and *in vivo*. The antibody shows potent inhibition of tumor growth in a broad spectrum of xenograft models in which ErbB3 is activated by its ligand NRG1 or by HER2 overexpression. AV-203 has recently started Phase I clinical development in 2012.

#### MP-RM-1

MP-RM-1 is a mouse monoclonal of undisclosed isotype generated by Mediapharma through repeated mice immunizations with NIH-3T3 cells transfected with the human ErbB3 coding sequence [90]. In spite of the weak affinity for the receptor (Kd 32 nM) this antibody is able to fully inhibit ligand-induced ErbB3/Akt activation in several cell lines in a time and dose-dependent fashion. The antibody is also able to induce receptor internalization and degradation, although with a slower kinetic than signaling inhibition. MP-RM-1 has been shown to block formation of ligand-induced heterodimers of ErbB3 with HER2, but is unable to compete with the ligand for binding to the receptor [90]. In the absence of a precise knowledge about the epitope recognized by this antibody it is possible to speculate that its binding to the receptor hinders the dimerization epitope or impairs the ligand-induced conformational change needed to expose the dimerization interface. MP-RM-1 has been shown to slow down tumor growth approximately 50% when given as single agent in two xenograft models tested. Perhaps the most interesting feature of this antibody is its ability to suppress ligand-independent activation of ErbB3. This was assessed in MKN-45 human gastric cancer cells, which do not express NRG-1β and betacellulin and where ErbB3 constitutive phosphorylation is triggered by association with cMet. MP-RM-1 was shown to fully inhibit pErbB3 in a manner similar to a cMet inhibitor and in co-immunoprecipitation studies was shown to inhibit association of ErbB3 with cMet [90]. This finding has important implications for the treatment of tumors where activation of ErbB3 is not ligand-dependent.

#### LMJ716

LJM716 is a fully human antibody isolated from a Morphosys phage library and being developed by Novartis and Sanofi Aventis [91]. LJM716 potently inhibits ErbB3/ Akt phosphorylation and proliferation in both ligand dependent and HER2 amplified ligand-independent ErbB3 dependent cell lines *in vitro*; it induces tumor growth inhibition or even regression, both as single agent or in combination with other ErbB receptor family targeted agents. LJM716 does not compete with NRG for binding to ErbB3. The crystal structure LJM716 bound to the ErbB3 extra-cellular domain revealed binding to a conformational epitope contained within domains 2 and 4 and trapping ErbB3 in the inactive conformation. LJM716 therefore possesses a novel mechanism of action; it prevents the structural rearrangements required for ErbB3 activation induced by either HER2 or NRG.

#### REGN1400

REGN1400 is a fully-human anti-ErbB3 monoclonal antibody under development by Regeneron Pharmaceuticals Inc. [92]. The antibody shows a very high affinity for the receptor (Kd approx 50pM) and a potent inhibition of NRG binding (IC_50_ approx 30pM). REGN1400 inhibits phosphorylation of ErbB3 and Akt in multiple human tumor cell lines and growth of these cell lines *in vitro*. REGN1400 strongly inhibits the growth of tumor xenografts in a dose-dependent manner both as single agent or in combination with anti EGFR or anti HER2 antibodies.

### Bispecific antibodies

#### MEHD7945A

MEHD7945A is a fully human dual specificity anti-ErbB3 and anti-EGFR monoclonal antibody under development by Genentech which was isolated from phage display Fab libraries [93, 95]. The objective of the authors was to obtain a wider spectrum monoclonal antibody capable of affecting ligand dependent proliferation of cancers which require signaling by EGFR or ErbB3 or both receptors simultaneously. The rationale behind the generation of this antibody was that, given similar structural configuration of the two receptors, their partial sequence homology and their similar mode of activation by ligand, it should be possible to obtain a normal IgG capable of interacting with either of the two antigens in their relevant ligand binding sites. The authors first isolated a monospecific anti-EGFR antibody from a phage library with diversity on the heavy chain CDRs. Then, by constructing a library with mutations in the light-chain CDRs, they were able to select clones that acquired binding to ErbB3 while maintaining binding to EGFR. MEHD7945A has a Kd of 1.9 nM for EGFR and of 0.4 nM for human ErbB3 respectively, is cross reactive with mouse EGFR but not with mouse ErbB3. MEHD7945A potently inhibits heregulin-induced pErbB3, pAkt and pERK 1/2 with IC_50_ values of 0.05, 0.19, and 1.13 μg/ml respectively. Likewise it inhibits TGFα induced pEGFR and pERK 1/2 with IC50 values of 0.03 and 0.16 μg/ml respectively. In *in vitro* proliferation assays in cell lines that are mainly dependent upon HER2-ErbB3 heterodimers (H1666) or upon EGFR-EGFR homodimers and EGFR heterodimers with other ErbB receptors, the antibody was more potent than monospecific antibodies such as cetuximab, pertuzumab or an anti-ErbB3 antibody.

The binding epitopes in EGFR and ErbB3 recognized by MEHD7945A were identified by X-ray crystallography of antibody receptor complexes. As expected they map in similar regions of domain 3, and overlap with the ligand binding site. However, the two epitopes are slightly shifted by a few Angstroms and are constituted by non homologous aminoacids. The binding mode of the antibody therefore suggests that its main mechanism of action is the block of ligand binding and fixing the receptors in a tethered conformation (Fig2).

MEHD7945A activity *in vivo* was tested in twelve xenograft models of various origin (NSCLC, ovarian, breast, pancreas, colorectal) in parallel with treatments with monospecific monoclonals against EGFR (cetuximab) or against ErbB3. In a subset of cases its activity was compared with the combination of the two monospecific antibodies. As expected from its mode of action MEHD7945A showed a broader profile and was efficacious in all lines tested: it had equal activity to cetuximab or to anti-ErbB3 in tumors which depend upon EGFR or ErbB3 respectively, and had higher potency than their combination in tumors which depend on both receptors. Interestingly, MEHD7945A is also able to induce antibody-dependent cellular cytotoxicity (ADCC) as shown in a series of *in vitro* and *in vivo* studies [93].

#### MM-111

MM-111 was rationally designed to be able to target tumor cells which acquire resistance to current HER2 inhibitors, such as trastuzumab or lapatinib. It has been observed that the mechanism of resistance is in most cases linked to ligand-dependent hyperactivation of ErbB3 in the presence of amplified-HER2 expression. In order to more efficiently target this type of tumors Merrimack pharmaceuticals recently generated MM-111 a bispecific antibody directed against both HER2 and ErBB3 [95]. MM-111 is made of two distinct single chain antibodies, B1D2 and H3 recognizing HER2 and ErbB3 with an affinity of 0.3 nM and 10 nM respectively, linked to a variant of recombinant human serum albumin (MHSA) with short connector linkers inserted at the amino and carboxy-termini of the molecule. MM-111 is able to engage simultaneously both receptors with the formation of inactive trimers and shows avidity binding to cells with an affinity for ErbB3 superior to that of the single sFv component (Fig.2). When tested *in vitro* in a panel of cell lines, MM-111 inhibits NRG induced pErbB3 and cell proliferation with an efficiency that correlates, as expected, with the degree of HER2 overexpression levels. MM-111 is stable in serum, *in vivo* upon injection in mice and shows prolonged terminal half-life. When tested as single agent in xenograft models its anti-tumor potency again correlated with the degree of HER2 expression, being most effective in HER2 +3 tumors at the dose of 30 mpk. Although MM-111 is directed against both HER2 and ErbB3 receptors, it is unable as single agent to fully block their activity. This can be indirectly inferred by the results of combination studies *in vitro* and *in vivo* with lapatinib or trastuzumab. Indeed combination of MM-111 with either lapatinib or trastuzumab in BT474 cells always showed superior efficacy. It is not clear why MM-111 alone is unable to fully inhibit HER2-ErbB3 activation, but one likely possibility is that this antibody does not trigger efficiently receptor internalization and degradation, This results in a residual receptor activation which needs additional targeted agents to be fully eliminated.

#### MM141

The evidence that a subset of cancer cell lines show simultaneous production of NRG and IGF1 and this gives rise to simultaneous activation of both receptors led Merrimack to generate a bispecific antibody directed against ErBB3 and IGF1R which was called MM-141 [96]. MM-141 blocks both NRG binding to ErbB3 and IGF-1/IGF-2 binding to IGF1R and inhibits downstream PI3K/Akt pathway activation (Fig.2). MM-141 may have the potential to be active on a wider spectrum of human tumors over a broad range of receptor profiles. More studies are however needed to precisely understand its superiority to monospecific anti-ErbB3 antibodies or dual specificity anti ErbB3-EGFR or anti-ErbB3-anti-HER2 antibodies.

## CONCLUSIONS

In recent years ErbB3 has emerged as a key player in the establishment of malignancy. Disregarded for several years largely because of the absence of detectable ErbB3 mutations in cancer samples and for the lack of a functional kinase domain in its intra-cytoplasmic region, ErbB3 is instead one of the most potent activators of the PI3K/Akt axis and is upregulated and trans-phosphorylated in several forms of cancer, in particular following treatment with EGFR and HER2 inhibitors. Hence ever growing evidences point to ErbB3 as an important factor in the establishment of resistance to therapies.

The most effective strategy to specifically target ErbB3 is by use of monoclonal antibodies. The number of anti-ErbB3 antibodies in development is impressive and unprecedented (see Table 1). This clearly underscores the importance this target has reached during the past years and the high expectation about their therapeutic impact.

Most importantly anti-ErbB3 monoclonals may become “universal” ingredients in “combination cocktails” conceived to attack tumors resistant to small molecule inhibitors or monoclonal antibodies directed against other EGFR family members [97]. Indeed it has been shown that both in tumors resistant to trastuzumab or in tumor resistant to gefitinib, ErbB3 is upregulated and, most importantly pErbB3 is constitutively activated. Therefore in these cases anti-ErbB3 antibodies able to strongly induce receptor internalization and degradation and to affect simultaneously both PI3K and ERK pathways activation may significantly sensitize resistant tumors to the action of HER2 or EGFR inhibitors, respectively.

These agents may have significantly different mode of binding, some of which are schematically represented in Figures 2 and 3. Figure 2 indicates the mode of action of some monospecific and dual specificity anti-ErbB3 antibodies. In Panel A a monospecific antibody binding to domain III inhibits ligand binding and blocks the receptor in a tethered configuration unable to form heterodimers with other receptors. In panel B the antibody (such as TK-A3) binds to the heterodimerization loop located in domain II. In panel C a dual specificity antibody such as MEHD7945A binds to domain III of two distinct receptors ErbB3 and EGFR. It is not excluded the possibility of formation of heterotrimers. In panel D a dual specificity antibody MM-111 interacts with its two scFv domains simultaneously with both HER2 and ErbB3. Panel E shows the mode of binding of a dual specificity antibody against ErbB3 and IGF1R such as MM-141.

Figure 3 shows how antibodies capable of triggering strong receptor internalization and degradation can act a strong therapeutic agents by wiping out this key signaling receptor from cells. This is for example what we have observed with TK-A3 and TK-A4 [86]. Furthermore, in addition to these direct effects it must be considered the possible indirect contribution of the immune system through ADCC for which agents such as GE-huMab-HER3 and MEHD7945A have been optimized.

With some distinct features, all the antibodies possess the biochemical properties and the therapeutic effects in preclinical models desired for a anti-cancer product. Obviously it is difficult to believe that all these agents will become marketed agents. Therefore, the race to which of them will be the winner(s) is entirely open. This will certainly depend upon the speed in advancing these agents to the clinic. However, a major role will be played by the strategy adopted in clinical development:

- will it be possible to identify suitable biomarkers for the selection of responders?
- will it be more convenient to target ligand-dependent tumors or ligand-independent ones?
- and in both cases which will be the best combination agent and tumor type to target first?

These are still open questions which are the main focus of current research and for which we hope answers will be found soon.
